# Individual (Non) Resilience of University Students to Digital Media Manipulation after COVID-19 (Case Study of Slovak Initiatives)

**DOI:** 10.3390/ijerph20021605

**Published:** 2023-01-16

**Authors:** Hedviga Tkácová, Martina Pavlíková, Eva Stranovská, Roman Králik

**Affiliations:** 1 Department of Journalism, Faculty of Arts and Letters, The Catholic University in Ruzomberok, 034 01 Ruzomberok, Slovakia; 2 Department of Journalism, Faculty of Arts, Constantine the Philosopher University in Nitra, 949 74 Nitra, Slovakia; 3 Department of Romance and German Studies, Faculty of Arts, Constantine the Philosopher University in Nitra, 949 74 Nitra, Slovakia; 4 Department of Russian Language, Peoples’ Friendship University of Russia (RUDN University), 117198 Moscow, Russia

**Keywords:** digital media, risk factors, false resilience, youth, positive proposal

## Abstract

The starting point of this theoretical article is the presentation of the issue of media manipulation in the contemporary digital media environment. The theoretical part is followed by a description and analysis of selected factors that create a belief of the individual’s resilience to digital media manipulative elements. Among the seven researched factors of an individual’s (non) resilience to digital media manipulation, we include: media illiteracy/literacy, thought activity/laziness, searching/not searching for “consensus” in the media, not emphasizing/emphasizing emotions, non-reliance/reliance on own intuition, non-credibility/credibility in the opinion of celebrities and automatic distrust/trust of recipients in the information presented in the media and others. We do not see the presence of manipulative elements in digital media as the main danger (manipulation is always in a sense part of the media message), nor the fact that manipulative elements have an effect on individuals (media—manipulative and non-manipulative—effects cannot be doubted). In our opinion, the very significant risk is the fact that the media message is followed by a false belief of the recipient’s own “immunity” against (covert and overt) media manipulation. The result of this false notion is the individual’s belief that manipulation in digital media content does not “affect” him (“I can easily recognize media manipulation”) and does not “touch” him (“It can’t happen to me”). Such a person then resembles a “house on the sand”, as his opinions, arguments or beliefs quickly collapse under the onslaught of five skills: challenges in the context of media literacy, critical thinking, strategies for verifying the credibility of information sources, the rational assessment of issues and reflection of reality. This original research article is a qualitative analysis of the legacy of ten Slovak educational initiatives focused on the issue of education in the context of media manipulation. The analysis focuses on the role of individual risk factors associated with resilience. The conclusion of the analysis is the elaboration of a positive proposal for the researched issue.

## 1. Introduction

At the end of the 20th century, the global media system literally took over the Internet, which created an environment for “democratic media communication” [1,2,3,4]. In today’s online environment, another specific group of media has become digital media and within this group, for example, is disinformation media, understood as media that produces content intentionally produced to mislead others. Despite many positives, the Internet environment brings many manipulative campaigns that are preplanned, cheap, effective, executed across multiple platforms and, at the same time, reaching a wide audience [5,6,7]. Manipulation on the Internet represents a certain way of influencing an individual or a group, or even an entire community, with the aim of changing their opinions and attitudes without them realizing it. Media manipulation chooses methods and tactics, as a result of which the manipulated person is convinced that he has made his own decision, i.e., he is convinced that it is a decision of his own free will. The aim of such manipulation is to mislead people. It tends to be more difficult to detect because it often represents a formal truth, although its content is actually a lie [8,9]. The core of media manipulation is therefore a danger that deserves our attention, especially because the increase in manipulative content is directly related to the current distrust of a large part of the population towards facts, expert information and scientific authorities [10,11,12,13]. The decline in these types of trust on a global scale is confirmed inter alia by a global study, which was carried out in 2021 by the Edelman Trust Barometer. This executive summary presented the conclusions that were related to the peak of the COVID-19 pandemic. The conclusions of this extensive research point out that, in addition to the respondents’ mistrust of facts, expert information and scientific authorities presented above, the danger is also an increase in manipulative content in society, which is accompanied by a high distrust of respondents, and even a crisis of public confidence in the responses of authorities to social challenges associated with the pandemic [14].

We must add that a significant change did not take place in 2022. The massive level of mistrust of people in most countries affected not only attempts to fight against COVID-19, but also an attempt to fight against media manipulation, misinformation or conspiracy thinking in relation to COVID-19 [15,16,17,18]. Other experts have therefore pointed out that there has been a visible shift from the “information age” to the “disinformation age” after the COVID-19 pandemic [19,20,21]. Similarly, experts from Slovakia since then—richer in experience with the pandemic and its consequences—warn, inter alia, against the low media literacy of the recipients [22], against the incomplete understanding of the educational potential of digital technologies [23], against not taking into account the individual and original abilities of students in the education process [24], about the bad habits of the recipients due to the use of media [25], about the presence of hoaxes in public media broadcasting [26], about the atmosphere of distrust in the disinformation web sites [27], about educational insecurity [28], etc. As a result of these trends, media manipulation and its manifestations are perceived as a global threat [29]. As we can see, the COVID-19 pandemic brought many negative consequences, which, in addition to the health care crisis (including mental health crisis), the economic crisis, the war crisis and the humanitarian crisis, include the full manifestation of the crisis of the “information age”.

Currently, also being discussed as one of the many consequences of several lockdowns, is the either reduced or limited access to education or changed forms of education in many countries, which switched to a distance form of education. This change during the pandemic took place in more than 190 countries and had an impact on more than 90 percent of all students in the world (1.57 billion young people) [30]. It was clear to all of them that the Internet became a significant and decisive tool in the management of distance education [31]. Students were forced to stay at home and use media in the educational process, i.e., media as a tool, such as computers and smartphones, as well as digital media, such as search engines (Google+), educational platforms (Yahoo), communication platforms (Teams), social networks (Instagram) and online news (Internet media). Similarly, some researchers identified three categories of how students used e-learning tools and adapted to e-learning during the pandemic: popular services and applications, popular applications for synchronous meetings and several others synchronous and asynchronous e-learning methods [32].

On a regional and even global scale, children and young people became the most vulnerable group during the COVID-19 pandemic [33,34,35,36,37,38]. Moreover, according to research, the amount of time young people spend on the Internet has increased recently [39]. Livingstone and Helper’s findings even remind us that older teens engage in more online communication activities than younger children do and so encounter more online risks. Similarly, young people at university age are in a period when risk and risky behavior is present and definitively confirmed [40].

Likewise, research confirmed that during the lockdowns, while teaching was eminently online, students presented higher levels of stress, loneliness, overwork, time pressure or difficulty in learning, and, for example, lack of wellbeing, the loss of the feeling of the community of learning, lower motivation or satisfaction with an academic result and so on when compared to other phases of teaching (i.e., hybrid and face-to-face phases of teaching) [41,42,43]. However, in this context, there are positive research findings that confirm significant changes in the attitudes towards distance education from the very beginning of the pandemic to their relative stabilization later [44].

Today we ask about the consequences, because it turns out that today’s young people (especially students and young workers) have been found to have an increase in risk factors, such as a feeling of loneliness, procrastination, losing their vitality and activity, increased alcohol consumption, addiction to computer games or the Internet, non-resilience to media manipulation and non-resilience to online disinformation, etc. [33,37,45,46,47,48,49,50,51]. The latter is the subject of our interest in this study. In our opinion, a significant risk is the false belief among the group of today’s youth, about their own “immunity” to (covert and overt) media manipulation, i.e., the belief that media manipulation does not “affect” them (“I will easily recognize every fabrication”) and does not “touch” them (“I will not believe any lie”). Thus, we study the new vulnerabilities of digital media users through media manipulation in this article.

Through the Internet, it is possible to find several very good websites that deal with the issue of resilience to digital media manipulation. Thomson Reuters foundation, for example, is also interested in media manipulation and resilience to digital media manipulation, and rather than debunking misinformation after it is already spread, the web emphasizes the need for prebunking, i.e., “preemptive debunking” of misinformation, which is described as “effective at reducing susceptibility to misinformation at scale” [52]. Another example of initiatives against misinformation represents the reactions of the European Union to misinformation and the manipulation of information. We are referring to the website called EUvsDisinfo, which also fights against misinformation through resilience guidelines, or the so-called EU Code of Practice in the field of disinformation, the key objective of which is, among other things, to ensure that purveyors of disinformation do not benefit from advertising revenue [53]. In the context of Slovakia, several websites also deal with the issue of resilience to digital media manipulation. First of all, the current government launched several projects to debunk COVID-related disinformation, and secondly, in May 2022, the Slovak parliament hastily passed an amendment to the Cyber Security Act. This amendment allows the National Security Authority to shut down sources of “malicious content”, i.e., software or data that cause cyber security incidents, fraud, theft of data, serious misinformation and many other forms of current hybrid threats [54].

Similarly, there are several studies that have examined the resilience of students to different variables. The research on students’ experiences of their individual resilience and their efforts of individual resilience is related, for example, to the goal of achieving the mental wellbeing of students against adversity and supporting their mental wellbeing [55,56,57]. Another group of studies has the characteristics of literature reviews [58], systematic reviews [59] or meta-analysis [60,61,62] as well as principle-based approaches [63]. Another group of studies focuses on the resilience of students to adverse living conditions or situations; in the last few decades, many of these have focused on the relationships between resilience, academic burnout, psychological health or wellbeing [64,65,66,67]. Most recent studies have narrowly focused on the situation related to COVID-19 or the war in Ukraine [68,69,70].

Recently, the number of studies examining the resilience of students has increased [71,72,73] although most of these have primarily focused on students’ resilience during the COVID-19 pandemic [74,75,76,77,78] and so on. The research findings and conclusions of those studies can be considered as the academic and civil society’s contribution to resilience. Their authors have directly engaged in information monitoring as well as strengthening society’s media and information literacy, and called for a combination of efforts by volunteers from the IT, civil society organizations, media, academia, education, governments and business sectors. They provide a reminder that strategies that focus solely on the repression of certain types of content are not sufficient as they are, at the very least, limited by the speed and dynamic nature of digital dissemination channels. Complementary prevention strategies that strengthen an individual’s resistance against digital manipulation are thus inevitable. As we can see, despite the obvious positives and real benefits of using different media as learning tools [79], it can be stated that the negative effects on the mental health of youth as presented above, and of digital media manipulation, have become, inter alia, significant signs of the COVID-19 pandemic.

On the other hand, there are only a few foreign studies that have primarily focused on students’ resilience against digital media manipulation after the COVID-19 pandemic, including research carried out in the post-pandemic era. With a focus on Slovakia in particular, this text and research is one of the first attempts to approach the issue of resilience against media manipulation after the COVID-19 pandemic. This case study of Slovak educational initiatives, based on qualitative analysis and focused on the issue of education in the context of media manipulation, can even be considered as a pilot study in our local context. Because prior findings do not apply to the current study, which is focused on the period after the COVID-19 pandemic in our local context, the current examination adds new insights to the extant research findings.

We study the new vulnerabilities of digital media users through media manipulation in this article. The main interest of our article focuses on university students and their subjective belief of their (more or less false) resilience to media manipulative elements. The first aim of this research is to identify the factors that create the student’s belief in their resilience to media manipulative elements. The result of this false notion is the individual’s belief that manipulation in media content does not affect him. Hence, the young digital media user is creating a more or less presumed image of self-resilience. The analysis focuses on the role of individual risk factors associated with resilience. The second aim of this research study is to point out the significance of challenges in the context of media literacy, critical thinking, strategies for verifying the credibility of information sources, the rational assessment of issues and (at least minimally) the reflection on reality. The conclusion of this paper provides the elaboration of a positive proposal in order to present possible reactions to the researched issue and its findings.

## 2. Characteristics of Selected Slovak Educational Initiatives Focused on Fight against Media Manipulation

We understand media literacy as the ability to be able to use and create information and be able to critically assess true and false information. The issue of media literacy in terms of the ability of pupils and students to detect media manipulation is a relatively new topic in Slovakia and it is not yet anchored in the content of school education. Despite that, it is possible to see rare initiatives that reflect the increase in manipulative tactics in digital media in Slovakia [80]. We consider further research in this area to be necessary.

As our study focuses on digital media, the researched educational initiatives originated and operate in the digital media environment. All are available in their online form. The list of researched initiatives that are the subject of this research is given in Table 1.

- Infosecurity.sk—This portal was created in response to a new situation created by a non-transparent information space of digital media. Infosecurity.sk is, among other things, an educational platform that clearly and effectively communicates key topics [81];
- AFP Facts—The AFP agency launched its fact-checking service in France in 2017. Through its website and Facebook page, it now offers the public monitoring of online content in several languages, i.e., takes into account the local context, culture, language and political setting of specific countries [82]. Information not only describes the source of the conspiracy and its dissemination in detail but also the number of those who disseminate information on social networks;
- Young people against fascism—the founder of the website (then a 16-year-old high school student) Marek Mach realized that in Slovakia (i.e., 37 years after the fall of totalitarianism) there is no website that contains information on extremism and fascism, and which would also communicate content understandable to young people. The website, including the Facebook page, provides information on historical events, which are often distorted in the media; above all, it clarifies the facts about the president of the (fascist) first Slovak state Jozef Tiso, it clarifies the position of the Roma during the Second World War and it describes the situation of Jews at that time, etc. [83];
- The Slovak Spectator—is the largest news portal about Slovakia in English [84]. The portal was included in the list of authorities due to its active interest in clarifying popular hoaxes and false reports in the European media (this is premium content) [85];
- Children on the Net—is a web portal focused on responsible digital parenting, which provides users with methodological material entitled “Learning to use technology safely and responsibly with children” [86]. The guide consists of six parts: children and mobile, children and the Internet, children and television, 10 principles for good “digital parenting”, answers to responsible parent’s questions and a dictionary. The portal focuses on two target groups, parents and teachers, although its readers may also be the general public, as it contains a variety of topics and news items from the area of the responsible digital existence of children and youths;
- Wolves—is a project that brings together experts in innovative education, especially editors, experts and fans of meaningful games and technologies that deserve the attention of parents and teachers. The project includes lectures for teachers, lecturers and parents, in which the possibilities of using modern technologies and games, including their educational potential and aesthetic value, in contemporary education are clearly and intelligibly presented [87];
- Sheep—is a fairy-tale series and its portal and methodological material are entitled “How not to lose a child in the world of the Internet: a handbook for teachers.” The initiative in Slovakia was established in 2009 and its goal is to raise awareness of the risks and dangers associated with the use of mobile phones and new technologies [88]. The series, portal and methodological material for educators are a response to the pan-European trend of “moving prevention to younger children so that they are informed about the threats of virtual space before they encounter them” [89];
- Hoax—is a Slovak educational website providing information about media manipulation with a closer focus on the issue of misinformation, disinformation, hoaxes and political social propaganda in online media space;
- Choose info—is a Slovak educational website providing valuable information about media manipulation as well as about building critical thinking. The portal includes a “Guide to social networks” in which you can find a description of the nature of the most famous social networks, and an overview of their positive and negative features, including tips on how to exist in social networks and how to approach them responsibly and safely [90];
- Handbook “Lies and Conspiracies”—is a handbook for high school students, which was created on the initiative of journalists and experts of the Slovak opinion-forming newspaper “Denník N”. The guide explains, based on concrete examples, how thousands of Slovak users disseminate and like misinformation content. The handbook contains specific examples used by readers to practice identifying manipulative tactics in media and digital media, suggestions for exercises with pupils in education, as well as practical explanations of the main types of media manipulation with concrete and actual demonstrations [91].

## 3. Materials and Methods

### 3.1. Research Design

Our goal is to analyze the legacy of ten educational initiatives presented above in Slovakia. The first goal is the identification factors that, according to experts, create the belief of individual’s resilience to media manipulation elements. The second goal is the identification of the most important skills that, according to experts, are needed to build real resilience to media manipulation in digital media.

As we can see in both cases, the opinions of experts who have created content of these ten educational initiatives is interesting to us, as they have focused for several years on the issue of youth education in the context of media manipulation in Slovakia.

The study contains three research questions:

O1: What factors cause young people’s (false) belief in their resilience to media manipulation?

O2: What are the most important abilities of young people that are needed to build their real resilience to media manipulation in digital media?

O3: What would a positive proposal for a system of education look like that would lead young users of digital media to the recognition of media manipulation and better handling of media manipulation?

### 3.2. Qualitative Data Collection

The research took place from March to October 2022. The content analysis was intended to solicit information regarding “risk factors” that create the belief of individual´s resilience to media manipulation elements (C1), and solicit information regarding important skills that are needed to build real resilience to media manipulation in digital media (C2).

The research sample for our research consisted of websites and several types of text documents, which are considered “materialized means of social communication that constitute a rich source of data for scientific research” [92]. Our research sample contained the following types of text documents: research texts (e.g., research reports, expertise), development texts (projects, expertise), texts with an educational and awareness focus (texts aimed at the development of national education, action programs), statistical overviews (statistical findings), administrative texts (minutes from meetings of the investigated authorities) and media reports.

We used a content analysis method on 160 selected texts that were created by the researched educational initiatives and published on their official websites. Research sample consisted of sixteen articles from each of ten researched initiatives. The analysis of the text material was directed from the collection of the data to conclusions (i.e., to a theory); the research was inductive in character, i.e., it was text-driven.

We used a content analysis within research method, because it is considered “an important methodological tool in the empirical knowledge and practical transformation of reality” [92] and it is applied in all research procedures in which one works with a written text (e.g., analysis of websites or questionnaires) also with the spoken word (e.g., interview analysis). Content analysis serves to analyze data and interpret their meaning [93], and it is valued for having the potential to provide results not only at the level of description and quantification of variables, but also by offering interpretative and explanatory conclusions [94,95], including explanations of social reality and social phenomena [96].

Thanks to this characteristic of content analysis, many researchers have applied this method in various areas and to a wider range of phenomena [97]. We mention, for example, the use of content analysis to measure the difficulty of didactic texts [98], to research media communications [99], to research value stereotypes in advertising [100], to analyze variable data and interpret their meaning [93], to study and collect data from written communication [101] as well as in different areas of sociology [102], for management research [103], oral communication research [104] and so on.

Examined educational initiatives focused on the issue of youth education in the context of media manipulation in Slovakia are presented above; i.e., in Table 1.

### 3.3. Procedure

All research items were first checked by the authors for their relevance for the study and then hand coded by four trained coders, who were fluent in both languages (i.e., Slovak and English), and were familiar with the Slovak media system. The full coding scheme is available upon request.

Within O1, the coding took place in a total of ten main categories. We noticed, for example, what the experts from the researched initiatives wrote about media illiteracy/literacy, thought activity/laziness, searching for/not looking for “consensus” in the media, not emphasizing/emphasizing emotions among users, and so on. The most discussed categories within 160 examined articles are presented in the Section 4.

Additionally, within O2, the coding took place in a total of ten main categories. We noticed what the experts from the researched initiatives said about the necessary abilities of young people to build real resilience to media manipulation in digital media, i.e., what experts wrote about the need for critical thinking, mastery of basic strategies for verifying the credibility of information sources, what they say about media literacy, etc. The most discussed categories in 160 examined texts are presented in the Section 4.

The scoring procedure employed was to add one point for each item. For example, if there was one specific “risk factor” experts pointed out in text, the researched initiative received one point. In fact, we assumed, that articles could have more than one researched risk factor each.

## 4. Results

As we assumed, there were more than 160 variables found within the 160 research texts during the coding. In fact, the research sample consisted of 307 variables within O1, and 173 variables within O2. In the next section, we present the research results based on the three specified research questions.

### 4.1. Factors That Give the False Belief of Individual Resilience to Media Manipulation Elements (O1)

In this article, we researched the factors behind the supposed “resistance” of an individual to media manipulation that takes place in digital media.

We used a content analysis method within 160 selected texts that present researched educational initiatives on their official website to research the most significant factors. During the coding, we found that experts focused on several problems in their articles, i.e., they wrote about several users´ “risk factors”. For this reason, we noticed that there were more than one main topic, i.e., in each text we identified from one to three risk factors. Thus, we categorized and coded a total of 307 variables.

**Figure 1:**
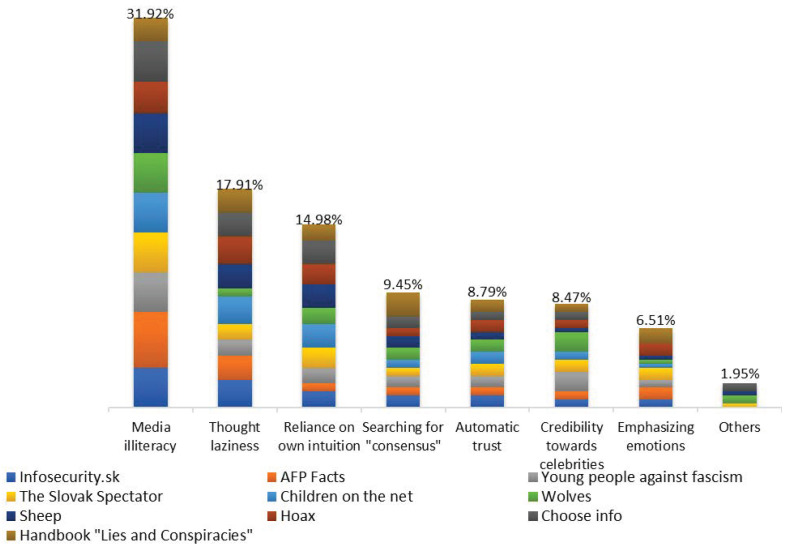
Research of risk factors of (false) resilience to media manipulation elements (n = 307).

We presented the identified factors as “risk factors” for young users that lead these users to believe the media content in the digital media environment despite the fact that it has manipulative content. According to experts, the (false) belief of students about their levels of resilience to media manipulation is mainly caused by seven factors. Our findings can be seen in Figure 1.

As we can see in Figure 1, within the content analysis of the ten initiatives, we identified seven factors, understood by experts understood to be “risk factors” for young people’s (false) resilience to media manipulation in the digital media environment. Based on 160 research texts with 307 variables, the order of “risk factors” from the most significant to the least significant is as follows: (1) media illiteracy (31.92%); (2) thought laziness (17.91%); (3) reliance on own intuition (14.98%); (4) searching for “consensus” in the media (9.45%); (5) automatic trust of recipients in the information presented in the media (8.79%); (6) credibility in the opinion of “celebrities” (8.47%); (7) emphasizing emotions (6.51%); and (8) others (1.95%).

We consider the content analysis of the 160 selected texts from these research initiatives as an appropriate research technique in order to make inferences by interpreting and coding textual material.

### 4.2. Most Important Skills Needed to Build Real Resilience to Media Manipulation in Digital Media (O2)

Based on the analysis of the legacy of the ten researched educational initiatives focused on the issue of the education and training of youth, we present five important skills, which, according to the experts and authors of the researched initiatives, are necessary to build young users’ real resilience to media manipulation within digital media.

**Figure 2:**
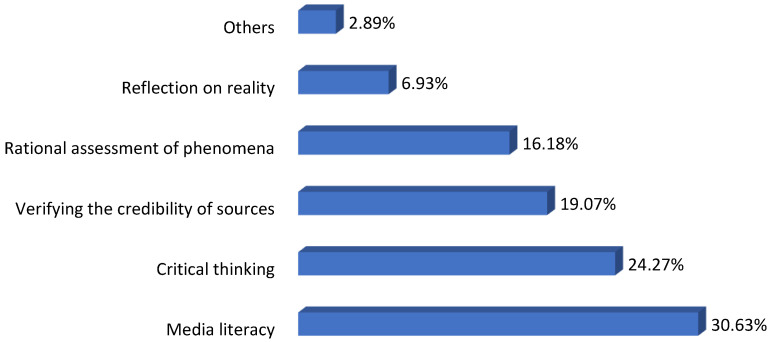
Research of the most important skills for building users´ real resilience (n = 173).

During coding, a similar situation occurred as in the first part of the research. Therefore, we again noticed that there was often more than one main skill emphasized within the researched text, i.e., in each text we identified from one to four skills. In the second part of our research, we categorized and coded a total of 173 variables within 160 research texts. Our findings are shown in Figure 2.

As we can see in Figure 2, with the use of the content analysis method, we found, according to the experts, the five most significant individual skills of young users in terms of using and being resilient to digital media: media literacy (30.63%), critical thinking of the individual (24.27%), mastering the strategy of verifying the credibility of information sources (19.07%), rational assessment of phenomena (16.18), the ability of at least minimal reflection on reality (6.93) and other skills (2.89%).

### 4.3. Presentation of a Positive Proposal of System of Education That Would Lead Users of Digital Media to the Recognition and Better Handling of Media Manipulation (Empirical Example from Slovakia) (O3)

According to Kačinová, the issue of educating students with a focus on media literacy in Slovakia, especially in the area of pupils and students’ ability to detect media manipulation tactics, is a relatively new topic; this is why it is not yet fully anchored in the content of school education [80]. Therefore, the topic of media manipulation is not explicitly mentioned in any thematic unit of the focal teaching subjects in Slovak schools, and it can be implicitly seen only marginally, as a part of other topics in the compulsory education system. In addition, the author draws attention to the fact that the education of and building of media literacy for students in Slovak schools is focused only within the context of print media, i.e., digital media are on the fringes of educational topics at both explicit and implicit levels. For this reason, we have thought about a positive proposal for a system of education that would lead users of digital media to the recognition and better handling of media manipulation.

We propose several needs:

1. The need to include topics related to media manipulation among compulsory educational topics—the inclusion of topics related to media manipulation (such as media manipulative tactics, past and present misinformation, conspiracy media, the fight against misinformation, etc.) in the educational content of compulsory subjects. The first subproposal is a response to the findings of a survey that monitored the countries of Central and Eastern Europe, including Slovakia, and confirmed that up to 56% of Slovaks believe in conspiracy theories, which is the most among the monitored countries. Experts associate the credibility of Slovaks in relationship to misinformation primarily with shortcomings in education, i.e., they talk in particular about the lack of incentives that would encourage children and young people to increase their critical thinking in education [105];
2. The need for education about media manipulation through parallel educational initiatives—the insufficient inclusion of topics related to media manipulation into compulsory education can be partially helped by parallel educational initiatives, which, in cooperation with schools, may represent specific activities of teachers or educational institutions. At their initiative, welcome educational projects, seminars and campaigns, including accompanying information materials, may emerge; we are referring, for example, to manuals, guides, brochures or newsletters that would briefly introduce students to media manipulation, media manipulation tactics, misinformation and key aspects of the fight against misinformation. In such a way, topics related to media manipulation can bring different kinds of benefits to students and can build the longer lasting media literacy of students;
3. The need for topics related to media manipulation as a part of teachers´ education—Current and effective implementation of the topic in the context of teaching is not possible without the support of teachers. In order to incorporate the topic of media manipulation as an educational topic, it is necessary to create methodological tools for the implementation of further teaching, which would be a reliable, erudite and unified tool for all teachers. We believe that a parallel phenomenon of this part of the proposal would be caution and greater resilience to media manipulation among teachers as well as a decrease in the credibility of the conspiracies, which was recorded in a huge amount among Slovak teachers in the recent survey [106];
4. The need to improve teacher’s practice of critical thinking—Teacher beliefs about the role and importance of critical thinking are also meaningful and this topic need its own research across various contexts; for instance, teachers may decide to demonstrate contradictory or even dangerous beliefs in the school class due to media manipulation. Improving teacher’s practice of critical thinking needs to be accomplished through instruction and other types of professional development for teachers even in the university level; neither age nor education are automatic prerequisites for a person’s critical thinking [107]. Teachers trained in these skills definitely become better teachers, which in turn can lead to them training students who become critical thinkers;
5. The need for the development of teachers’ beliefs as an important “unobservable cognitive dimension”—The third and fourth points are related to the positive proposal, which take into account not only the need for teacher education but also the continuous development of a teacher’s beliefs, i.e., their belief as an “unobservable cognitive dimension” [108] (p. 81) of teaching and learning. The fact is that the topic of a teacher’s beliefs has been receiving increasing attention in the field of teacher education [109,110,111]. In this context, several researches have been conducted. For example, research on in-service teachers’ beliefs towards teaching methodologies [112], preservice teachers’ evolving beliefs about and perceptions of themselves [113], the relationship between teachers’ stated beliefs and practices [114] and so on. Moreover, several experts pointed out that it is the mental state of the teacher that guides his thinking processes as well as his activities, and it has a significant effect on his behaviors and classroom practices, or, for example, his instructional choices. Therefore, attending to the beliefs of teachers should be a focus of educational researchers;
6. The need to use dialogic-based education and dialogic-based assessment in education about media and media manipulation—Given the importance of the depth of knowledge that students should be able to not only demonstrate but also use in daily life, in the context of media manipulation, the use of dialogic-based assessment in education seems to be very helpful. This kind of assessment repeatedly confirms, for example, the improvement in students´ learning through active settings or collaborative settings, which are understood to be interactive and, at the same time, as group or team education activities (e.g., [115,116]), and where dialog between teacher and students, as well as between students, is the main part of the education process. In this process, social interaction is such a key element. Dialogic-based formative education consists of the use of active communication between the teacher and student and features a positive way for a student to be able to express their opinions and new observations in an accurate and logical manner. Moreover, dialogic-based assessment seems to be a suitable approach in education because the teacher acts as a motivator and promoter who simultaneously provides immediate feedback during the whole education process. More importantly, the focus of dialogic-based education and assessment is on students’ future development and practice [117,118], which is important due to the rapid development of media communication and its consequences and effects on human life;
7. The need for understanding media education and its benefits in relation to societal negatives—Last but not least, education about current forms and tactics of media manipulation (preferably in the form of the inclusion of these topics within the compulsory educational topics and therefore as early as possible in the development of the individual) could also be beneficial in the context of different societal negatives. Several experts in Slovakia pointed out, for example, about one Slovak specific. They associated current societal negatives in Slovakia with the “historical memory of the nation”. According to experts from the Center for Social and Psychological Sciences of the Slovak Academy of Sciences, Slovaks are subject to media manipulation relatively quickly because it follows from their recent history, which was dominated by the belief in “quick and simplified theories” that people have created to understand the surrounding and often too complex (and hurtful) reality. Gulisová states that Slovaks have a “historically rooted attitude they gained from the past when they had to endure communist censorship” [119]. The communist regime taught people not to trust official information, which partly affects, for example, the current confidence of Slovaks in misinformation. After all, this is also the reason why we—as a starting point —propose to include the issue of media manipulation in the educational content of compulsory educational subjects to a sufficient extent and as soon as possible.

The last part of our research intends to offer insights into the theory and practice that underlie effective education against manipulation within digital media. Several points from the positive proposal (C3), as specific empirical examples from Slovakia, have ambitions to be motivations for leading digital media users as to the gradual removal of “risk factors” and to contribute to the discussion about current post-COVID education.

## 5. Discussion

The issue of media manipulation in the current digital media environment, which we address in our study, is related to and agrees with the existing theory. As with the Thomson Reuters foundation, our findings also emphasize the need for prebunking, respective “preemptive debunking” of misinformation, which is described as “effective at reducing susceptibility to misinformation at scale” [52]. We are also convinced that other “resilience guidelines” such as the EU Code of Practice in the field of disinformation may be necessary because they help strengthen individual resilience against cyber security incidents, fraud, theft of data, serious misinformation and many other forms of current hybrid threats [54].

Because misinformation contributes to the creation of unfavorable living conditions (for example, as a result of the automatic trust of recipients in the information presented in the media or the automatic trust in people in the online space [88,89]; see Figure 1) or contributes to the creation of unfavorable situations (for example, by emphasizing emotions, such as anger or hatred, within the online space; see Figure 1), we strive, similarly to other authors, to provide a reminder that strategies that focus solely on the repression of certain types of content are not sufficient; they are, at the very least, limited by the speed and dynamics of digital dissemination channels. Complementary prevention strategies that strengthen the individual’s resistance against digital manipulation are thus inevitable. Through our own research, the current study adds new insights to the extant findings, focused on the research of Slovak educational initiatives. Our goal was to analyze the legacy of ten educational initiatives from Slovakia; in the Slovak context, it was a pilot study.

We consider the new vulnerabilities of digital media users as fundamental to the future of whole societies and their democracies. However, instead of political, economic or axiological perspectives, which have also become factors of “the objective wellbeing” [120], we decided to look for the possibility of a solution to media manipulation in the dialectic regarding the seven “risk factors” of users and also through the five identified user skills, and their application to current, we could say post-COVID, education.

In the following section, we discuss the results of our findings and interpret these findings. We found that there are seven main factors behind the supposed “resistance” of an individual to media manipulation. We present the identification of these researched factors in the form of two opposite poles. The analysis focuses on the role of individual risk factors associated with resilience.

1. Media literacy provides digital media users with: (a) a sovereign orientation in the various contents; (b) the ability to quickly distinguish essential and serious information from non-serious information, including the reliable identification of false information, incomplete information or inaccurate information; (c) the ability to distinguishing serious, verified message/statement from subjective content and unsubstantiated facts [121]; and (d) the ability to use as well as develop critical thinking, which is a prerequisite for prudent use of the media [122].

Conversely, the user’s risk factor is media illiteracy. According to several opinions that are voiced in the examined texts on the educational platforms, media illiteracy—in our research in 31.92% of people—fundamentally deprives users of at least five abilities already mentioned, and, at the same time, of the benefits resulting from a correct understanding of true as well as false media content.

2. Thought activity versus the risk factor of thought laziness—An important problem that the researched educational initiatives point out is the occurrence and influence of media manipulation tactics and its creators on the individual opinion of a person as well as the public opinion of society. Behind the problem of media manipulation in socially important topics is the efforts of its creators. However, it is the responsibility of users not to succumb to passivity, but on the contrary, to subscribe to an honest and active understanding of the problem. The warning comes not only from our findings, but also from the same observations of other experts outside of our research, who unanimously state that one has a natural tendency to believe that something is true, although it is not, if one does not have to think about it very much [123].
3. Non-reliance versus the risk factor of reliance on one’s own intuition—Every user, as the recipient of a message, has a major influence on how he personally perceives, evaluates and trusts the information. The ten initiatives in our research pay considerable attention to understanding the psychology of Internet news consumption among young users. In addition to the phenomenon of a user´s credibility, most of the researched initiatives also pay significant attention to the key internal process of the individual during his contact with media manipulation. According to experts in the researched initiatives, relying on one’s own intuition when in contact with media manipulation can be considered as a disadvantage for the individual in the process of assessing the nature of the information. The problem of the initial intuitive assessment of information (and, subsequently, the rational assessment of information) is that it tends to lead a person to quick conclusions even without relevant supporting arguments. In addition, being controlled by the original emotion that is a part of manipulative information (most often alarming false information) encourages the rapid and mass spreading of these messages in the digital media space.
  Conversely, based on the content analysis of the selected initiatives, (non) reliance on one’s own intuition in assessing the veracity of information is considered a key feature, but one on which it is necessary to exert effort. While intuition is defined as a preliminary opinion, estimate or idea of truth that results from a mostly momentary feeling (a sense of clarity, a sense of certainty) [124], thinking that does not rely on one’s own intuition is considered an everyday challenge, according to our research. In the context of the initiatives examined, this challenge is a call for the individual to think, which would be the result of cognitive reality, i.e., the result of clearly formulated rational reasons.
4. Not searching versus the risk factor of searching for “consensus” in the media—Based on his research, Nickerson confirms that trust in false reports can be built when false reports confirm existing attitudes, beliefs or hypotheses that an individual believes or trusts. The author says that a person is even “biased” in seeking confirmation of his reasoning [125]. Experts within the ten researched educational initiatives further agree that searching for “consensus” in digital media, on the first hand, creates a community and deepens interpersonal relationships with those with whom we find “common language”. However, on the other hand, there is a risk of an environment existing in which one ideological or cultural perspective is presented and this perspective excludes or misrepresents all other interpretations of the issue. Representative research realized among students in Central Europe comes to the same conclusion when confirming that, for example, manipulative and false content is always a concern, especially when they are a significant reason for the creation and permanence of intentional manipulations inside of homogeneous groups operating in and through digital media [126]. Finally, an individual’s dependence on their existence in a social network environment, according to the experts, is linked to the individual’s dependence on the content he or she likes [90].
5. Automatic distrust versus the risk factor of the automatic trust of recipients in the information presented in the media—The credibility of different kinds of information, as well as its acceptance on both an individual and social level, appears to be a current problem, especially in the area of digital media, which are based on the idea of a free and collective approach to online content that is created by users. According to research, in the new media environment, the most vulnerable group is young people, whose time on the Internet has recently increased the most [127]. The trustworthiness and naivety of children and young people is considered a “child risk factor” with regards to the Internet because, in the online environment, “children do not attribute bad intentions to others” [88]. Thus, they cannot know who they can trust in a virtual environment [89]. We therefore consider the false notion of young people that they can estimate the level of trust in relation to other people in the digital media space as a risk factor.
6. Non-credibility versus the risk factor of credibility in the opinion of “celebrities”—The credibility of information and news in relation with peoples´ changes in attitudes and behaviors has been studied for decades in the persuasion literature. However, how we define and understand the credibility of information changed when users of digital media increasingly became creators of online content, including manipulative content [128,129]. There are no longer experts, but instead there are new celebrities who have experiences that they show to and share with the public. These celebrities reflect individual values, and these values, in practice, become not only the mechanism of the behavior of an individual, but also, in a broader sense, of the way in which elements of culture are organized [130]. According to Leslie, a celebrity is defined by three basic characteristics: acquaintance, frequent appearances in the media and the long-term maintenance of this status. The importance of a celebrity usually determines the permanence of the public’s interest in the personality [131]. The experts consider young people’s trust in the views of “celebrities” to be a modern phenomenon, and in accord with them, in this article, we consider this to be a risk factor for digital media users.
7. Not emphasizing versus the risk factor of emphasizing emotions—The Internet environment seems impersonal to many people, giving the impression that it is not real and that practically everything can be in it. Hence, in an effort to eliminate the negatives of online communication (invectives, hate speech, rumors, fake news and hoaxes and so on), it is important to focus on education about netiquette (etiquette on the Internet), and even on appropriate behavior in the online environment [88]. In addition, excessive emphasis on emotions (especially anger and hatred) or the use of invectives is a violation of human and social boundaries, according to experts from the ten researched initiatives; in this sense, excessive emphasis on one’s own feelings and emotions can be understood as a risk factor for young users. Moreover, youth need to be made aware that events in the virtual environment, including emotional expressions or emotional outbursts, have an impact on people in the real world; according to the experts these might affect, for example, people´s subjective satisfaction, wellbeing or the happiness of individuals with their own lives [132].

We do not see the presence of manipulative elements in digital media as the main danger (manipulation is always in a sense part of the media message), nor the fact that manipulative elements have an effect on individuals (media—manipulative and non-manipulative—effects cannot be doubted). In our opinion, the very significant risk is the fact that the media message is followed by a false belief of the recipient’s own “immunity” against (covert and overt) media manipulation. The current study highlights this and adds new insights to the extant findings, focused on the research of Slovak educational initiatives. We should know what the “risk factors” are that are associated with the resilience of students after COVID-19, and we have to know how to gradually remove these “risk factors” in order to contribute to the growth and improvement in the current post-COVID education system based on current needs. In our opinion, this is the way to create conditions for the development of the individual (non) resilience of university students to digital media manipulation after the COVID-19 pandemic.

Speaking about false resilience to media manipulation among today’s university students, first of all, according to the experts from the researched initiatives, it is important to educate youth in their ability to use at least a minimum of skills associated with building media literacy. According to the analysis, it is also necessary to educate young people mainly in critical thinking and being able to verify the credibility of information sources, and the in rational assessment of the phenomena that young people are reading about, are watching or are listening to. Similarly, the following research findings are a challenge for current, we could say post-COVID, education. Its goal must be to create the conditions for the motivation of a new generation of critical thinking people, who have the ability to use their skills associated with media literacy, who attempt to ask appropriate questions, who seek relevant information, who reason in an organized way, who make comprehensive conclusions, who reflect on the information, who assess the creditability of information, etc. Let us add that, according to the experts, two individual attitudes of young people are important: (a) willingness of the individual’s thought commitment (i.e., willingness to think) and (b) willingness of the individual’s self-education (i.e., willingness to self-educate).

Lastly, we introduced the positive proposal for a system of education that would lead users of digital media to the recognition and better handling of media manipulation. The positive proposal was formulated through seven subproposals that reflect the topic of media manipulation as being an educational topic within the content of the compulsory education in schools in Slovakia because education focused on media literacy in connection with media manipulation is not yet enshrined in the content of school education in Slovakia [80]. Hence, the proposal points to some needs: (1) The need to include topics related to media manipulation among compulsory educational topics. They should be included in the educational content of compulsory subjects as soon as possible, and thus be of benefit to students as a their part of compulsory education and in terms of building students’ critical thinking [105]. (2) The need for education about media manipulation through parallel educational initiatives. (3) The need for topics related to media manipulation to be a part of teachers´ education, i.e., methodological manuals and, at the same time, educating educators in the field of media manipulation, misinformation, conspiracies, etc. [106]. (4) The need to improve teacher’s practice of critical thinking. (5) The need to develop teachers beliefs as an important “unobservable cognitive dimension” [108] of teaching and learning processes. (6) The need for the use of dialogic-based education and dialogic-based assessment in education about media and media manipulation to improve students’ future development and practice [117,118]; and (7) the need for this to be of benefit to the whole society, i.e., the historical memory of the Slovak nation [119]. The third part of our research offered insights into relevant theory and practice that might provide ideas for the generation of motivating and effective education for young people regarding information manipulation in the current digital media.

Relevant research, the views of the experts from the ten initiatives, as well as our own research findings confirm that the set of seven factors and the set of five user skills introduced in this article, might be considered as important parts of the current education, and which call for their rapid application to the educational process. We consider the research findings to be a cognitive benefit of the present study.

## 6. Limitations

Qualitative content analysis has proven itself for a long time as effective in the analysis of media content in various scientific fields [133], as it enables a more complex and deeper understanding of media content, and the research of basic argumentative categories that are present in media texts is also a benefit of the chosen research method [134]. However, regarding the limitations of this study, we consider it important to briefly mention the methodological challenges in qualitative content analysis, especially the level of abstraction and the degree of interpretation used in constructing categories, descriptive themes and themes of meaning, which is related to the credibility and authenticity of the analysis [135]. In order to better manage these challenges, we implemented a simple and clear research design based on three research questions, and chose these for qualitative data collection. Finally, all research items were first checked by the authors for their relevance for the study. All four authors further jointly established the coding categories and agreed on the coding rules. All items were checked by the authors firstly, and secondly, their relevance for the study was hand coded but with a bigger number of trained coders.

Speaking about the content analysis, we do not want to forget the limitations of this research but another research method that could collect research data and supplement our research findings could be included. We would welcome, for example, a structured questionnaire that would examine two of our research variables from the perspective of users—students. Hence, it might be interesting to compare our research findings (content analysis method) with the opinions of students (questionnaire) on the issue of “risk factors” that create students’ belief about their resilience to media manipulation elements (C1). Equally interesting could be research findings on the issue of important skills that are, according to respondents, needed to build their real resilience to media manipulation in digital media (C2).

Likewise, the just mentioned research limitations might be considered as suggestions for future research. Of course, it is necessary to implement a trial phase, which is essential in the process of creating and formulating the research questions in the questionnaire. Likewise, a previous validation study could be helpful for researchers during the verification of the accuracy of the measurements.

Secondly, research using content analysis could also be replicated to analyze whether the results of other research converge with the results of this research. That is also why this research can be considered an exploratory study that has the ambition to contribute to the discussion about “risk factors” that create the belief of an individual’s resilience to media manipulation elements (C1), and to the discussion about the important skills that are needed to build real resilience to media manipulation in digital media (C2). It can be stated that other research, as well as the future faster adaptation of research outcomes into practice, can help advance both.

Our final suggestion for future research depends on teachers. If our goal is to provide not only effective but also long-term solutions, the consistent involvement of teachers in the formulation of new education proposals in the post-pandemic era seems necessary. Therefore, future research that focuses on the current possibilities of teachers’ intervention in education and accepting new proposals in the post-pandemic period seems equally useful. We believe that such research can reveal the possibilities of the greater intervention of educators in the present as well as new requirements in education and thus clearly name the places where the teacher’s work is necessary and irreplaceable.

## 7. Conclusions

After experiencing the pandemic and several lockdowns, people today can contemplate with satisfaction the new technological conquests and their application in many fields, especially in those dealing with human needs. In this context, the Internet and digital media have a truly positive and an irreplaceable place. On the other hand, the Internet is a widespread tool, a tactic and a favorite territory, integral to challenging the relations of media manipulation. We studied the new vulnerabilities of digital media users’ somewhere between these two poles.

The result of our research analysis of ten Slovak educational initiatives, focused on the issue of education in the context of media manipulation, was the identification of seven factors that cause the beliefs of university students about their individual resilience to media manipulation. We named these factors as “risk factors” for users because, according to experts, they are the result of an individual’s beliefs about their own resilience to media manipulation (and not the result of objective truth). Media manipulation therefore might bring “blindness” to media manipulation as well as unwanted trust among the current youth generation. Furthermore, within the content analysis of ten initiatives, we identified five skills that are the most important to educate young people in according to the experts. In the context of the research findings, a positive proposal was formulated, reflecting the topic of media manipulation as an educational topic in the content of the compulsory education in Slovakia.

The current examination adds new insights to the extant findings as there are only a few foreign studies (including research) that primarily focus on students’ resilience against digital media manipulation after the COVID-19 pandemic. This case study of Slovak educational initiatives, based on qualitative analysis and focused on the issue of education in the context of media manipulation, is the first such study in our context. We believe that the identified factors and skills, including several points that make up the positive proposal leading to the gradual removal of users’ “risk factors” (7) (i.e., empirical example from Slovakia), might play an important role in the current context of education, and, parallelly, also have a key role in changing society in Slovakia, which went, inter alia, through rapid and dramatic social, cultural and economic changes after the fall of the former regime (i.e., communism). However, as Gaffney and Holmes rightly point out, it is necessary to be vigilant because the “new” is still indelibly marked by the “old” [136]. Accordingly, it seems that rapid development can also be a pitfall if the “old wounds” are not healed, or, as the famous poet and novelist A. S. Pushkin said much more precisely, if the old wounds are not healed, also daylight goes out (paraphrase of A. S. Pushkin’s poem “The daylight went out”, 1820) [137].

We can take the same lesson from the COVID-19 pandemic, which has been characterized by many media manipulations that not only affected a large number of users and young users, but are still being continuously applied today through digital media. Hence, speaking about the individual (non) resilience of university students to digital media manipulation after COVID-19 (based on the case study of Slovak initiatives), as apparent as it seems to be, in digital media space is significant. There needs to be (1) education in the field of media literacy, which enables one to acquire the ability to critically analyze and evaluate media content [138] and (2) the necessity to apply educational methods in daily practice, i.e., to contribute to the habit of critically examining media content as well as expand students’ abilities to correctly interpret and critically evaluate media content on a daily basis [139]. We consider the individual resilience of students to digital media manipulation as a significant area of current research as well as being a question of human empowerment, development and investment in the future.

## Figures and Tables

**Table 1:** Examined educational initiatives focused on the issue of youth education in the context of media manipulation in Slovakia.

Researched Educational Initiatives	Source
Infosecurity.sk	https://infosecurity.sk (accessed on 1 March 2022)
AFP Facts	https://fakty.afp.com/list (accessed on 1 March 2022)
Young people against fascism	https://www.mladiprotifasizmu.sk (accessed on 1 March 2022)
The Slovak Spectator	https://spectator.sme.sk/t/3198/hoax (accessed on 1 March 2022)
Children on the net	https://www.detinanete.sk (accessed on 1 March 2022)
Wolves	https://vlcata.sk (accessed on 1 March 2022)
Sheep	http://www.zodpovedne.sk/download/metodika_OVCE.pdf (accessed on 1 March 2022) http://sheeplive.eu/sites/default/files/prirucka_ucitelia_a4.pdf (accessed on 1 March 2022)
Hoax	www.hoax.sk (accessed on 1 March 2022)
Choose info	https://zvolsi.info/sk/https://zvolsi.info/app/uploads/2020/01/zvolsi_brozura_digitalni.pdf (accessed on 1 March 2022)
Handbook “Lies and Conspiracies”	https://a-static.projektn.sk/2017/04/dennikN-prirucka-konspiracie.pdf (accessed on 1 March 2022)

## Data Availability

The data presented in this study are available from the authors upon request.
